# Preference for Different Anchor Descriptors on Visual Analogue Scales among Japanese Patients with Chronic Pain

**DOI:** 10.1371/journal.pone.0099891

**Published:** 2014-06-13

**Authors:** Junya Yokobe, Masaki Kitahara, Masato Matsushima, Shoichi Uezono

**Affiliations:** 1 Department of Anesthesiology, The Jikei University School of Medicine, Minato, Tokyo, Japan; 2 Division of Clinical Epidemiology, The Jikei University School of Medicine, Minato, Tokyo, Japan; The University of Tokyo Hospital, Japan

## Abstract

**Context:**

Although many previous studies have examined the preference of patients for different pain measurement scales, preference for anchor descriptors has not been thoroughly discussed.

**Objectives:**

To examine (1) the preferred end-phrases used in the VAS as anchor labels for Japanese patients with chronic pain, and (2) whether the preference differs according to factors such as age, sex, educational level, duration of pain, and pain intensity.

**Methods:**

We performed an observational study in patients suffering from non-cancer chronic pain for more than 3 months at a pain center in Japan. The patients were asked to rate their pain intensity using four types of VAS that used the following different anchor descriptors: “worst pain” (“Worst”), “worst pain bearable” (“Bearable”), “worst pain imaginable” (“Imaginable”), and “worst pain you have ever experienced” (“Experienced”). They were also asked to rank the four scales according to ease of responding, and asked which descriptor best reflected their perceived pain.

**Results:**

In total, 183 patients participated in the study. They consisted of 119 (65.0%) women and 64 (35.0%) men aged 18–84 years with the mean age of 56.9 years. “Experienced” was most preferred (69.8%), followed by “Bearable” (66.3%), “Worst” (48.8%), and “Imaginable” (16.9%). Factors such as age, sex, educational background, duration of pain, and pain intensity did not significantly affect the results. In 83.1% of patients, the preferred descriptor corresponded to the descriptor that best reflected patients' perceived pain.

**Conclusion:**

The frequently used expression “worst pain imaginable” is considered to be difficult to understand for most patients. Widely preferred descriptors, such as “worst pain you have ever experienced” and “worst pain bearable”, should be used when evaluating perceived pain. The preference of anchor descriptors was not significantly affected by the factors such as age, sex, educational level, duration of pain, and pain intensity.

## Introduction

The International Association for the Study of Pain (IASP) states that “Pain is always subjective. Each individual learns the application of the word through experiences related to injury in early life” [1]. Pain is thus an individual experience with multi-dimensional aspects [2]–[4], and, theoretically speaking, pain intensity is impossible to measure objectively and quantitatively [5]. However, accurate quantitative evaluation of perceived pain intensity is a prerequisite for both clinical practice and research purposes [5]–[8].

Over the past few decades, several self-reporting unidimensional pain scales have been developed, such as the Visual Analogue Scale (VAS), Numerical Rating Scale (NRS), Verbal Rating Scale (VRS), and the Faces Pain Scale. Of these scales, the VAS and NRS have been used most frequently worldwide because of their outstanding simplicity, and their reliability and validity have been proved [6]–[10]. These scales are designed to measure pain intensity using extremely painful conditions or situations as a point of reference. However, many descriptors have been used as anchor labels with the VAS and NRS in Japanese as well as other languages, and these descriptors have not yet been standardized [11]. Pain intensity cannot be precisely compared without using standardized anchor descriptors, even if the same pain measurement scale is used.

Several studies have investigated the preferred pain assessment tool among the VAS, NRS, VRS, and so on [8], [12]–[16]. However, very few researchers have paid attention to the descriptors used as anchor labels. Many studies have recommended the use of the NRS [6], [12], [17]–[25] and VAS [14], [26]–[28] to evaluate pain intensity; nevertheless, patients are sometimes unable to use these scales to correctly rate their perceived pain intensity in clinical and research settings. Some studies have shown that categorical scales such as the VRS are preferred over the VAS or NRS by elderly patients [2], [8], [14], [29], less educated patients [13], cognitively impaired patients [30], and children [31]. These results might be due not only to difficulties with abstract thinking [2], [30]–[32] but also to the ambiguity of anchor descriptors used in the VAS or NRS. Because appropriate quantitative evaluation of patients' pain intensity requires the patients to be able to obtain a clear image of the situations described in the anchor label, investigation of preferred anchor descriptors is expected to contribute to the improvement of pain intensity evaluations.

The two major purposes of this study are to examine (1) the preferred end-phrases used in the VAS as anchor labels for Japanese patients with chronic pain, and (2) whether the preference differs according to factors such as age, sex, educational level, duration of pain, and pain intensity.

## Methods

### Participants

The subjects for this study were recruited from chronic pain patients who attended the multi-disciplinary outpatient pain clinic of the Jikei University Hospital between June 2012 and May 2013. Most of the patients who attend the clinic were suffering from multiple types of chronic pain. The clinic consists of doctors, nurses, physiotherapists, clinical psychologists, and acupuncturists. The patients were basically treated with medication, therapeutic exercise, psychophysiological approach, and acupuncture. Inclusion criteria were a diagnosis of chronic non-cancer pain with a duration of more than 3 months and age 18 years or older. Patients who had visual disturbance, were unable to speak Japanese, or were suspected to have impaired cognition (Mini-Mental State Examination (MMSE) score < 23 points) were excluded.

### Anchor descriptors

The following four commonly used descriptors of the anchor labels used in the VAS in Japan were selected.

“worst pain” (hereafter “Worst”)

“worst pain bearable” (hereafter “Bearable”)

“worst pain imaginable” (hereafter “Imaginable”)

“worst pain you have ever experienced” (hereafter “Experienced”)

An American psychologist who was proficient in Japanese validated the translations of anchor descriptors used in Japanese to make sure that they were consistent with the expressions used in English [11].

### Questionnaire

A traditional VAS with a straight, horizontal 10-cm line was used, with the anchor label “no pain” at the left side and one of the translated Japanese descriptors at the right side. The four different VASs were placed on a sheet of paper in fixed order. The participants were asked to mark a vertical line on each VAS corresponding to their perceived average pain during the previous 24 hours. The participants were also asked to rank the scales in order of preference, from first to fourth place, on the basis of ease of responding, and they were also asked which descriptor best expressed their own pain.

### Ethics Statement

The protocol of this study was approved by the ethics committees of the Jikei University Hospital, and the study was carried out in accordance with the Declaration of Helsinki. Written informed consent was obtained before study entry from all patients who participated in the study.

### Data collection

The questionnaire written in Japanese was distributed to the participants when they gave consent after attending the pain clinic, and was collected on the same day. Age, sex, educational background, duration of pain, VAS, VRS, Hospital Anxiety and Depression Scale (HADS) [33]–[34], and Pain Catastrophizing Scale (PCS) score [35] data, which were administered on the same day, were obtained from medical records. Because the participants of this study are from the multi-disciplinary pain clinic, it is expected that their psychological factors have severely influenced on their pain condition. Therefore, we used HADS and PCS to verify that we could regard the participants to be the representatives of chronic pain patients in Japan. HADS includes two subscales which are anxiety and depression. The total score of each subscale is 21. The cut-off point of the each subscale of HADS is usually set as 8 and 11. The point between 8 and 10 indicates that the patient probably has anxiety or depression; the point of 11 and over definitely has anxiety or depression. The cut-off point of PCS is usually set as 30. The point of 30 and over indicates that the patient is in pain catastrophizing state.

### Statistical analysis

All data were analyzed statistically using the SPSS program (version 21.0, SPSS Inc., Chicago, IL, USA). Data treated as a continuous scale are presented as median and range, and categorical and ordinal data are presented as counts and percentages. Wilcoxon signed-rank test and chi­square test was used to compare the values and proportions between elderly and young group (Table 1). Chi­square test was applied to compare the proportions of preference between two groups (Table 2).

**Table 1:** General characteristics of study participants (n = 183).

	Total (n = 183)	Elderly (n = 87)	Young (n = 96)	p-value
Age, years	59 (18–84)	69 (61–84)	46 (18–60)	< 0.0001**
10–19	1 (0.6)			
20–29	9 (4.9)			
30–39	17 (9.3)			
40–49	33 (18.0)			
50–59	32 (17.5)			
60–69	49 (26.8)			
70–79	34 (18.6)			
80-	8 (4.4)			
Sex				0.42
Female	119 (65.0)	54 (62.1)	65 (67.7)	
Male	64 (35.0)	33 (37.9)	31 (32.3)	
Educational level				0.03*
Junior high school	12 (6.8)	7 (8.5)	5 (5.3)	
High school	79 (44.6)	44 (53.7)	35 (36.8)	
College/University	86 (48.6)	31 (37.8)	55 (57.9)	
Duration of pain, months	36 (3–480)	40 (3–240)	33 (3–480)	0.82
HADS (Anxiety)	7 (0–16)	7 (0–15)	7 (0–16)	0.31
HADS (Depression)	7 (0–20)	7 (0–17)	8 (0–20)	0.35
PCS	32.5 (7–51)	32 (9–51)	33 (7–50)	0.58
VRS				0.27
None	3 (1.7)	2 (2.3)	1 (1.1)	
Mild	62 (34.3)	25 (29.1)	37 (38.9)	
Moderate	96 (53.0)	45 (52.3)	51 (53.7)	
Severe	20 (11.0)	14 (16.3)	6 (6.3)	
VAS ("Worst")	59 (0–100)	65 (0–100)	55 (2–100)	0.27
VAS ("Bearable")	61 (0–100)	59 (0–100)	61.5 (2–100)	0.70
VAS ("Imaginable")	58 (0–100)	68 (0–100)	51 (0–100)	0.03**
VAS ("Experienced")	66 (0–100)	73 (0–100)	55.5 (0–100)	< 0.01**

Values are expressed as the median (range) or as number (percentage).

HADS = Hospital Anxiety and Depression Scale; PCS = Pain Catastrophizing Scale;

VRS = Verbal Rating Scale; VAS = Visual Analogue Scale

* chi-square test statistically significant (p<0.05).

** Wilcoxon rank sum test statistically significant (p<0.05).

**Table 2:** Percentage of participants who considered each descriptor as preferable (sum of 1st and 2nd place).

	A ("Worst")	B ("Bearable")	C ("Imaginable")	D ("Experienced")	p-value
All (n = 183)	48.8%	66.3%	16.9%	69.8%	
Young (n = 96)	46.2%	66.3%	13.2%	75.0%	
					0.41
Elderly (n = 87)	51.9%	66.3%	21.0%	63.8%	
Low education (n = 91)	47.8%	60.4%	20.9%	72.8%	
					0.34
High education (n = 86)	50.0%	72.8%	12.3%	66.3%	
Female (n = 119)	52.2%	62.8%	17.5%	69.3%	
					0.74
Male (n = 64)	42.4%	72.9%	15.5%	70.7%	
Short duration (n = 94)	48.9%	66.7%	14.6%	71.9%	
					0.87
Long duration(n = 89)	48.8%	65.9%	19.3%	67.5%	
Low pain intensity (n = 65)	55.0%	63.9%	18.3%	67.2%	
					0.87
High pain intensity (n = 116)	45.3%	67.6%	17.0%	70.5%	

Elderly, older than 60 years; Young, 60 years or younger;

Low education, junior high school or high school only; high education, college/university or graduate school;

Short duration, thirty six months or less; Long duration, more than thirty six months;

Low pain intensity, sum of "none" and "mild"; High pain intensity, sum of "moderate" and "severe" of Verbal Rating Scale (VRS)

**Figure 1:**
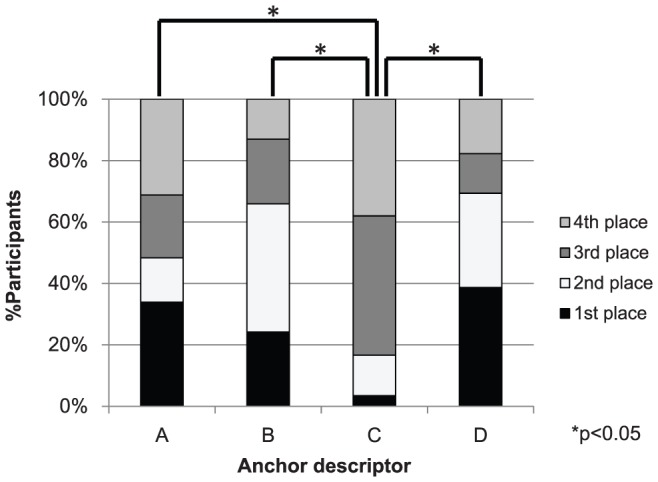
Preference of VAS with the four different descriptors. Friedman test demonstrated a significant difference between the four different descriptors (p<0.001); Bonferroni's post-hoc tests demonstrated significant differences between “Imaginable” and “Worst”, “Bearable”, and “Experienced” (p<0.05). A: worst pain (“Worst”). B: worst pain bearable (“Bearable”). C: worst pain imaginable (“Imaginable”). D: worst pain you have ever experienced (“Experienced”).

**Figure 2:**
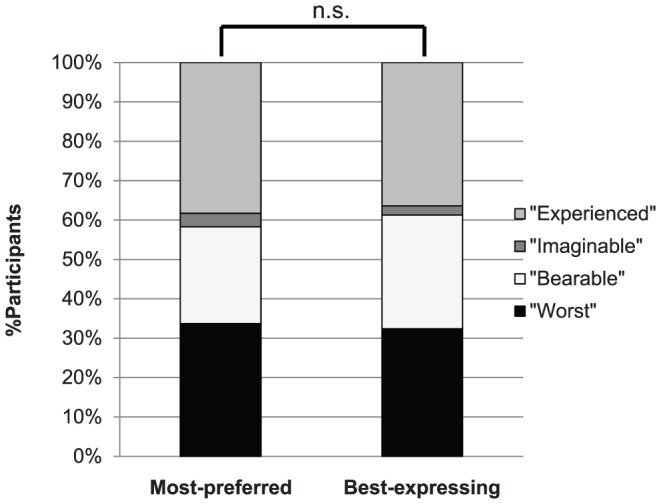
Comparison of percentages between the most-preferred and best-expressing descriptor. n.s., not significant; Wilcoxon's signed rank test, “Worst”: worst pain, “Bearable”: worst pain bearable, “Imaginable”: worst pain imaginable, “Experienced”: worst pain you have ever experienced.

Friedman test was used for analyzing the differences in preference among the different descriptors (Figure 1). Bonferroni correction was employed for *post-hoc* comparison of each pair. Wilcoxon signed-rank test was used for analyzing the relationship between the most-preferred descriptor and best-expressing descriptor (Figure 2). Spearman's rank correlation coefficient was calculated to examine whether the reported VAS pain levels using the different anchor descriptors correlate with each other (Table 3). Wilcoxon signed-rank test of the VAS values between the most-preferred descriptor and the least-preferred descriptor, was also performed to examine if the reported pain level significantly differed depending on the anchor descriptor. P-values less than 0.05 were considered statistically significant.

**Table 3:** Pairwise Spearman's rank correlation coefficient between the pain intensity measured by VASs using four anchor descriptors.

	A	B	C	D
A ("Worst")	-			
B ("Bearable")	0.79*	-		
C ("Imaginable")	0.82*	0.78*	-	
D ("Experienced")	0.81*	0.77*	0.87*	-

* Spearman's rank correlation coefficient statistically significant (p<0.05).

## Results

One hundred and eighty-three patients gave consent to participate in the study. Table 1 shows the demographic characteristics of study participants. Almost all of the participants were able to answer the VASs with four different descriptors; only four participants failed to mark at least one of the four VASs. The HADS score suggested that 27.9% of the respondents were in a depressive state, and 26.8% had anxiety. The PCS score indicated that 60.1% of the respondents were catastrophized.

Table 4 shows the characteristics of the participants' pain (some patients have multiple nature of pain). The majority of the participants are suffering from some of musculoskeletal pain.

**Table 4:** Characteristics of pain (n = 183).

	n
Diagnosis	
Myofascial pain	132
Peripheral neuropathy	19
Spinal degenerative disease	19
Sacroiliac joint dysfunction	18
Failed back syndrome	12
Post-operative pain	9
Postherpetic neuralgia	8
Arthritis	7
Other spinal diseases	6
Complex regional pain syndrome	4
Fibromyalgia	3
Non-specific low back pain	3
Trigeminal neuralgia	3
Headache	2
Type of pain	
Nociceptive	185
Neuropathic	50
Mixed	31
Site of pain	
Face, head, and neck	44
Shoulder and upper extremities	40
Chest and abdomen	16
Back	25
Lumber and buttock	65
Inguinal region and lower extremities	60
Others	5

Figure 1 shows the rankings given to the four different descriptors used with the VASs in this study. “Experienced” was most preferred by the largest number of participants (38.5%), followed by “Worst” (33.9%), “Bearable” (24.1%), and “Imaginable” (3.4%). The descriptor ranked as least preferred by most participants was “Imaginable” (38.2%), followed by “Worst” (31.2%), “Experienced” (17.6%), and “Bearable” (12.9%). Friedman test demonstrated the differences among the four different descriptors to be statistically significant (p<0.001); Bonferroni *post-hoc* tests demonstrated significant differences between “Imaginable” and “Worst”, “Bearable”, and “Experienced” (p<0.05).

Figure 2 compares percentages between the most-preferred and the best-expressing descriptor. Overall, 83.1% of the descriptors were consistent with each other.

Table 2 shows the percentage of participants that ranked each of the descriptors first or second in terms of preference. “Experienced” and “Bearable” were regarded as preferable by 69.8% and 66.3% of the participants, respectively. Almost half of the participants regarded “Worst” as preferable; “Imaginable” was regarded as preferable by only 16.9% of the participants. As Table 2 shows, approximately the same percentage of preference was observed even when the participants were stratified by age, sex, educational level, duration of pain, and pain intensity, with p-values, analyzed by chi-square test, of 0.41, 0.34, 0.74, 0.87, and 0.87, respectively. Table 3 shows pairwise Spearman's rank correlation coefficient between the pain intensity measured by VASs using the four anchor descriptors. The Spearman's rank correlation coefficient indicated that there were strong correlations between each pair of the anchor descriptors. There was a significant difference between the VAS values with the most-preferred descriptor and the least-preferred descriptor, analyzed by Wilcoxon signed-rank test, with the p-value of 0.038.

## Discussion

The key findings of this study are that (1) Japanese patients with chronic pain show a certain preference concerning the anchor descriptors of extremes; (2) most participants found that one descriptor was both easy to answer and best described their own pain; and (3) preference is not significantly influenced by factors such as age, sex, educational background, duration of pain, and pain intensity of the patient.

### Comparison of preference among different anchor descriptors

We found in this study that Japanese patients with chronic pain show a certain preference concerning the descriptors of extremes (Figure 1). The participants preferred “Experienced” and “Bearable” over the other two descriptors, while “Imaginable” was preferred significantly less often than the other descriptors. “Experienced” represents the “real” pain that an individual has actually experienced; almost all individuals, except for some who may have forgotten episodes of severe pain, should be able to remember and imagine such pain. One plausible explanation for the high preference of the descriptors “Experienced” and “Bearable” is that they are relatively easy to relate to.

On the other hand, “Imaginable” required the participants to imagine unexperienced pain, making it difficult to create a concrete image. Some participants mentioned that rather than imagining unexperienced pain, they remembered pain episodes that they had actually experienced.

Interestingly, approximately half of the participants regarded “Worst” as preferable and half as not preferable. Some participants interpreted “the worst pain imaginable” to mean pain they had experienced, while others interpreted this to mean unexperienced pain. The IASP defines pain as “an unpleasant sensory and emotional experience associated with actual or potential tissue damage, or described in terms of such damage” [1]. “Worst” seems to involve affective and emotional aspects more than the other three descriptors, which may have influenced participants' preference.

We also found in this study that participants tended to regard one descriptor as both easy to answer and best to describe their own pain. We defined “preference” as easiest to answer in this study. Peters et al. found that there was little difference between the scales that patients regarded as best describing, “most easy”, and “preferred” [8]. Similarly, our results suggest that the anchor label that best described participants' own pain tended to be the one they answered most easily.

As Table 3 showed, the anchor descriptor did not have a great influence on VAS values of the whole group. However, the result of the Wilcoxon signed-rank test indicated that preference of the anchor descriptor could affect the intra-personal pain intensity. Therefore, the easiest descriptor for each patient to understand should be used to evaluate that patient's pain accurately. Moreover, we had probably better not to use the less preferred anchor descriptor, such as “worst pain imaginable” to obtain precise data.

### Comparison of preference by subgroups

Although Table 2 shows that participants who were female, younger, and had a low level of education tended to prefer “Experienced” to “Bearable” and, conversely, that male, older, and highly educated participants tended to prefer “Bearable” to “Experienced”, the effect of these factors did not reach statistical significance. Previous studies have found that sex influenced the preference of pain scales to a certain extent [8], [14]–[15]. In any case, the percentage of participants that considered “Experienced” to be preferable was similar to the percentage that preferred “Bearable”. On the other hand, few participants preferred “Imaginable”, and the rank of preference of “Worst” and “Imaginable” did not differ according to subcategories. One plausible explanation is that some patients may have difficulty with the abstract concepts of inexperienced pain such as “Worst” and “Imaginable”.

Although “Imaginable” is a frequently used descriptor in Japan, its use as an anchor label in pain measurement may not provide an appropriate evaluation, because “preference” was defined as ease in understanding or answering in this study. Therefore, we consider it more suitable to use descriptors such as “Experienced” or “Bearable” for the evaluation of pain intensity in Japanese patients with chronic pain.

Many previous studies have investigated the preferences of patients for pain assessment tools. However, to the best of our knowledge, very few studies have dealt with the preference for anchor labels. Hjermstad et al.’s review article concerning pain intensity assessment tools written in English reports the use of 24 different descriptors in 239 papers with the VAS and NRS [11]. Thus, a range of non-standardized descriptors are used to evaluate pain intensity both domestically and internationally. Our study indicates that the preference of patients for descriptors can determine how precisely patients are able to communicate their perceived pain intensity.

These results suggest that anchor descriptors should be standardized as soon as possible for more appropriate evaluation of pain intensity. The findings from this study can contribute to more accurate assessment of the intensity of chronic pain. It should be noted that the participants in this study were from a limited population and that factors such as race, ethnicity, and culture are also known to influence pain perception [36]. To what extent our results extend to patients from other racial and cultural backgrounds is unclear and requires further study.

### Study limitations

This study has several limitations. First, patients with other types of pain, such as acute pain and cancer pain, were not included. It remains to be clarified whether the findings from this study can be generalized to all Japanese patients.

Second, participants in this study attended a multi-disciplinary pain clinic, introducing a probable selection bias. However, the range of pain intensity measured by the VAS and VRS, as well as psychometric factors such as the HADS and PCS, were distributed widely, so the participants can be considered to represent common Japanese chronic pain patients.

Third, the meaning of the descriptors in Japanese might not necessarily correspond exactly to their English counterparts. However, as the translated descriptors were checked by an American psychologist who is proficient in Japanese, we do not consider this to be a serious problem.

## Conclusions

The result of this study indicate that “worst pain you have ever experienced” and “worst pain bearable” are appropriate descriptors for Japanese chronic pain patients. “Worst pain imaginable”, though it is frequently used worldwide, may be inappropriate for evaluating pain intensity, at least among Japanese chronic pain patients. Widely preferred descriptors, i.e., most patients can respond without difficulty, should be used when evaluating perceived pain.

The preference of anchor descriptors was not significantly affected by the factors such as such as age, sex, educational level, duration of pain, and pain intensity.
